# Xylazine Withdrawal: A Case Report From the Intensive Care Unit to the Medical Ward

**DOI:** 10.7759/cureus.87545

**Published:** 2025-07-08

**Authors:** Hsien Yi Yang, Cyrus Nensey

**Affiliations:** 1 Hospital Medicine, Northridge Hospital Medical Center, Los Angeles, USA

**Keywords:** acute pain management, fentanyl use, hypertension, intensive care unit, opioid withdrawal, personalized addiction medicine, psychomotor agitation, seizure, xylazine, xylazine withdrawal

## Abstract

Xylazine is a centrally acting alpha-2 adrenergic agonist commonly used with fentanyl in illicit drug mixtures, yet its withdrawal profile remains poorly characterized. We report a case of a 35-year-old man with polysubstance use, significant for fentanyl with xylazine and bipolar disorder. He presented with seizure-like activity and agonal breathing, requiring intensive care unit admission. The patient’s persistent hypertension, agitation, and autonomic dysregulation were inconsistent with typical opioid withdrawal or sepsis. Initial management with sedatives and multiple antihypertensives was ineffective. Agitation improved with dexmedetomidine, whereas blood pressure and autonomic control were achieved after initiation of a transdermal clonidine patch, followed by transition to oral clonidine with tapering. The adjunctive use of gabapentin, lacosamide, and quetiapine helped manage psychomotor agitation and generalized pain. The patient recovered to his baseline and was discharged to an outpatient rehabilitation program. This case highlights the importance of recognizing xylazine withdrawal as an independent toxidrome requiring targeted alpha-2 agonist therapy and supports evidence-based, multidisciplinary collaboration for symptom control and clinical stabilization.

## Introduction

Xylazine, also known as “Tranq,” is a centrally acting alpha-2 adrenergic receptor agonist that inhibits the release of norepinephrine and epinephrine, resulting in sedation, analgesia, bradycardia, hypotension, and respiratory depression [1,2]. Although it is currently approved for veterinary use in the United States, xylazine has increasingly been identified as an adulterant in illicit opioids such as fentanyl, commonly referred to as “Tranq Dope” [1,2]. Xylazine enters the illicit drug supply through diversion from veterinary sources and is commonly added to fentanyl powders by local dealers or suppliers to increase bulk, enhance perceived potency, reduce the frequency of injection, and prolong euphoria [1,2]. First reported in Puerto Rico in the early 2000s and later spreading through the Northeastern U.S. - particularly Pennsylvania, Maryland, New York, and Connecticut, where mortality rates were highest - xylazine has now been detected in 48 states [1]. Xylazine-involved poisoning deaths increased a thousandfold from 2018 to 2021, highlighting its significant role in the ongoing opioid crisis [1].

The use of fentanyl with xylazine was declared a national emerging threat by the White House in 2023 [1]. Despite its growing prevalence and recognized public health impact, clinical understanding of xylazine withdrawal remains limited, with no standardized treatment guidelines [2]. This case report describes the acute care course of a patient with xylazine withdrawal, highlighting diagnostic challenges and management strategies across both the intensive care unit (ICU) and the medical ward. It underscores the urgent need for clinical awareness and evidence-based practices as xylazine continues to pose a major public health concern in the United States.

## Case presentation

A 35-year-old man with a history of bipolar disorder and polysubstance use, including cocaine, cannabis, and fentanyl with xylazine, presented to the emergency department in May 2025 following an episode of seizure-like activity at a residential drug rehabilitation center. He had flown in from Pennsylvania to attend the program and had only been at the facility for a couple of hours when the event occurred. It was unclear whether he had used any illicit substances while at the facility, but he reported obtaining his fentanyl and xylazine supply from the street in Philadelphia. He was found unresponsive at the scene, and staff initiated cardiopulmonary resuscitation before paramedics arrived. The patient received four doses of naloxone en route. Upon arrival at our hospital, he was agitated and exhibited agonal breathing. He was given levetiracetam and lorazepam, intubated for acute hypoxic respiratory failure, and admitted to the ICU.

The patient had a longstanding history of stimulant and opioid use, as well as intermittent homelessness and unemployment. His mother reported a prior diagnosis of bipolar disorder, for which he had been prescribed aripiprazole; however, medication compliance was uncertain. He denied alcohol use and had no known drug allergies or prior surgical history.

**Figure 1:**
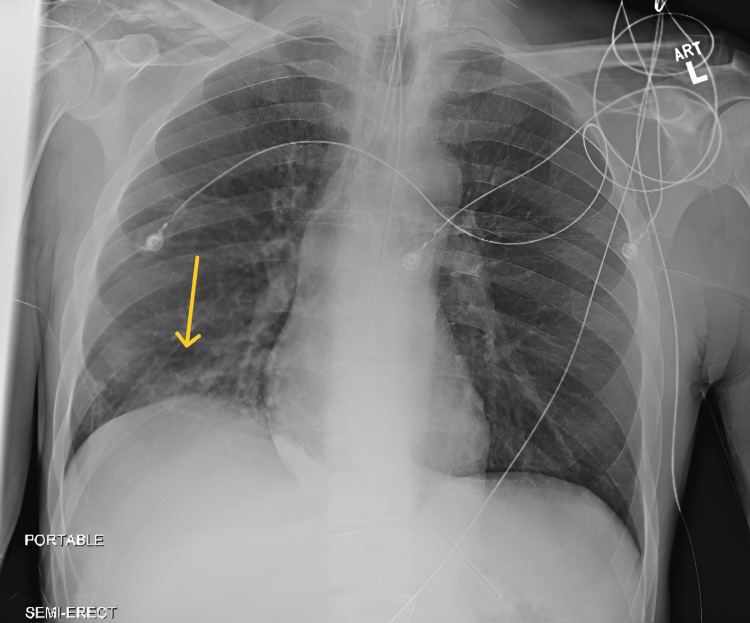
Chest radiography. The yellow arrow indicates basilar opacity.

**Figure 2:**
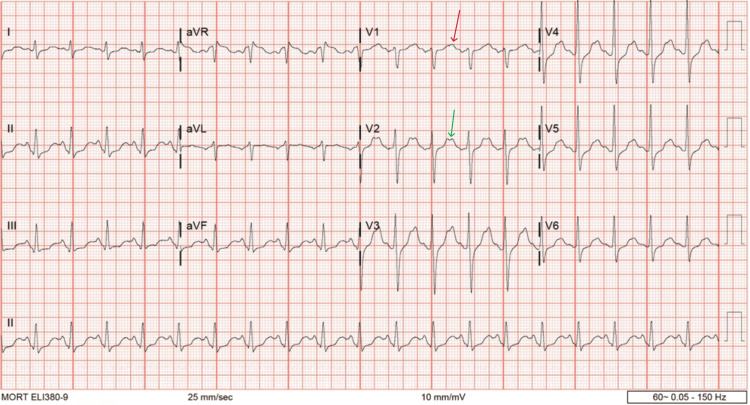
Electrocardiography reveals sinus tachycardia with biphasic T waves in leads V1 (red arrow) and V2 (green arrow).

On admission, the patient was hypertensive, with a systolic blood pressure (SBP) greater than 180 mmHg, and exhibited marked psychomotor agitation. He was sedated with continuous intravenous fentanyl and propofol. In addition, lorazepam and lacosamide were added for seizure-like activity. Quetiapine was administered to address agitation and underlying bipolar disorder. Chest radiography revealed basilar interstitial opacities, more prominent on the right (Figure 1). Electrocardiography (ECG) demonstrated sinus tachycardia with nonspecific T-wave changes (Figure 2). Laboratory studies revealed leukocytosis, elevated serum lactate, mild acute kidney injury (AKI), and hypokalemia (Table 1). He met criteria for sepsis and was treated for methicillin-resistant Staphylococcus aureus (MRSA) pneumonia with intravenous vancomycin.

**Table 1:** Laboratory investigations on the day of admission.

Investigation	Result	Normal Range
White blood cells (/uL)	16700	4000-11,000
Potassium (mEq/L)	2.5	3.4-5.0
Blood urea nitrogen (mg/dL)	23	8.0-21.0
Creatinine (mg/dL)	1.1	0.6-1.3
Lactic acid (mmol/L)	9.4	0.5-2.2

Although the initial clinical picture raised concern for infectious etiologies - supported by leukocytosis, elevated lactate, pulmonary infiltrates, and positive sputum cultures - the patient’s persistent hemodynamic instability, marked by persistent hypertension and agitation, was more consistent with xylazine withdrawal. Despite antibiotic therapy and improvement in respiratory status, he continued to exhibit SBP between 160 and 200 mmHg in the absence of fever or worsening leukocytosis. The hypertension was resistant to intravenous hydralazine and labetalol, oral losartan and amlodipine, and only partially responsive to dexmedetomidine infusion.

**Figure 3:**
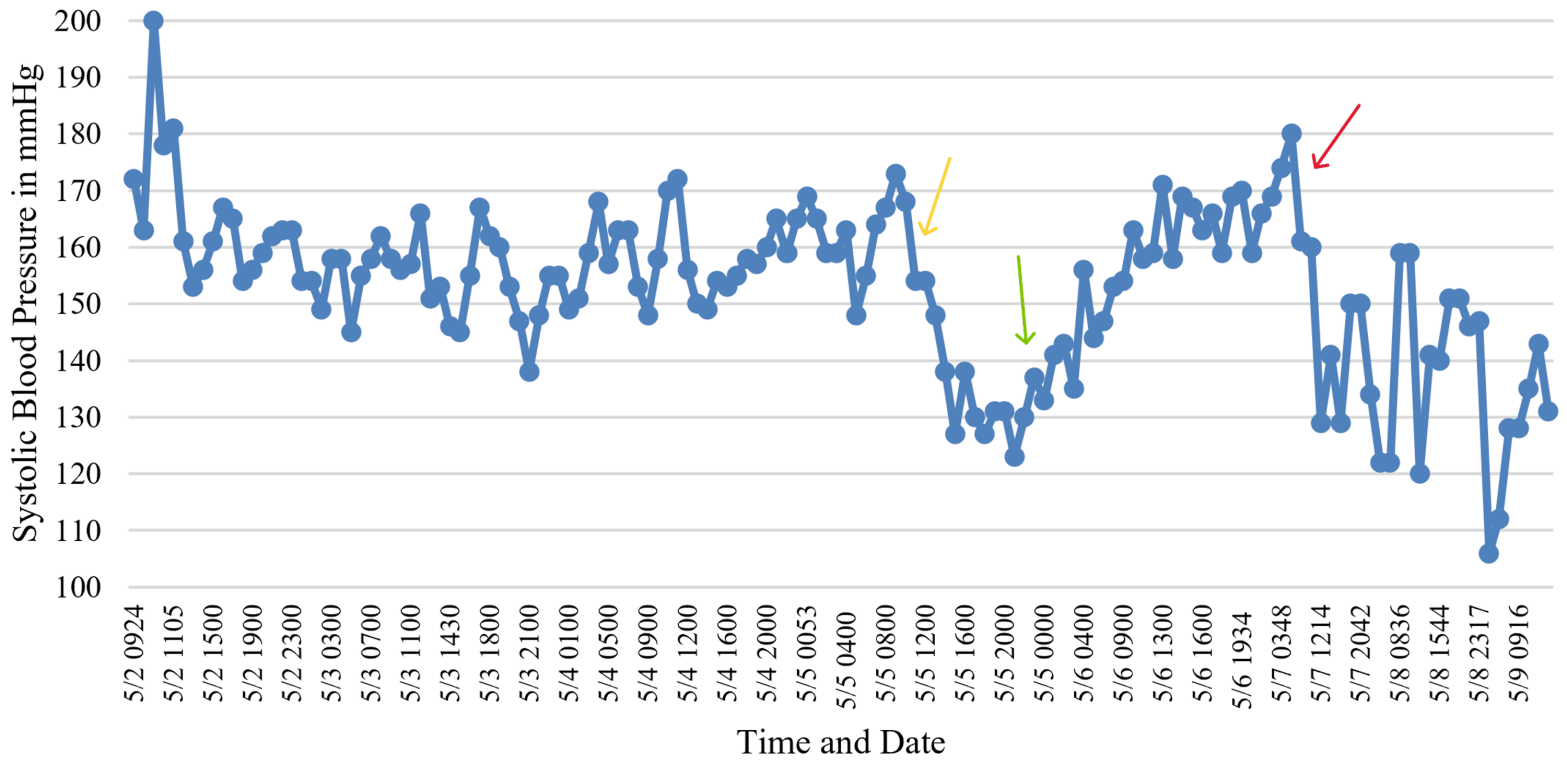
SBP throughout the hospital course. SBP decreased after clonidine initiation (yellow arrow). A transient increase followed dexmedetomidine initiation (green arrow). A further decline occurred after switching to oral clonidine (red arrow). SBP: systolic blood pressure

He received hydralazine in the emergency room, followed by losartan and amlodipine via nasogastric tube and intravenous labetalol in the ICU. On hospital day 1, SBP peaked at 200 mmHg. Elevated SBP persisted through days 2 and 3 despite dexmedetomidine therapy. On day 4, a transdermal clonidine patch was initiated, resulting in a sustained decrease in SBP to the range of 100 to 120 mmHg. The same day, following sedation weaning, the patient successfully completed a spontaneous awakening trial and a spontaneous breathing trial. He was extubated and transitioned from supplemental oxygen via nasal cannula to room air. On day 5, after passing a swallowing evaluation, the patient transitioned from the patch to oral clonidine, with continued improvement in SBP (Figure 3). Amlodipine was discontinued, and clonidine was gradually tapered. On day 6, gabapentin was initiated to address generalized pain and agitation, which facilitated the removal of physical restraints and improved patient participation in physical and occupational therapy. By hospital days 7 and 8, the patient remained normotensive on clonidine and losartan. He ambulated with a front-wheeled walker and tolerated a regular diet. Leukocytosis resolved, renal function and electrolytes improved, and oxygen requirements returned to room air (Table 2). The patient recovered to near-baseline mental status and was discharged to an outpatient rehabilitation facility.

**Table 2:** Laboratory investigations on the day of discharge.

Investigation	Result	Normal range
White blood cells (/uL)	5800	4000-11,000
Potassium (mEq/L)	4.9	3.4-5.0
Blood urea nitrogen (mg/dL)	16	8.0-21.0
Creatinine (mg/dL)	0.99	0.6-1.3
Lactic acid (mmol/L)	1.9	0.5-2.2

In short, rebound hypertension and agitation - despite sedatives and anti-seizure medications, and non-reversibility by naloxone - were key indicators distinguishing xylazine withdrawal from opioid withdrawal.

## Discussion

Coinjection of xylazine and opioids, particularly fentanyl, is an increasingly common feature of the current opioid epidemic [2]. As demonstrated in our patient, who tested positive for fentanyl with a known history of xylazine use, distinguishing between opioid withdrawal and xylazine withdrawal is essential to prevent misdiagnosis and treatment delays, especially when the xylazine use history is not clear. Moreover, opioid and xylazine withdrawal frequently occur simultaneously [2]. In patients with polysubstance use, it is crucial not to attribute altered mental status solely to intoxication or withdrawal, as other disease processes may be concurrent.

In this case, the patient’s leukocytosis and pulmonary infiltrates prompted an appropriate sepsis workup and stepwise antibiotic therapy. However, the presence of persistent hypertension - rather than the hypotension typically seen in septic shock - prompted consideration of alternative etiologies. Although the patient’s diagnosis of MRSA pneumonia was clinically significant, it did not fully account for the neurovegetative symptoms and autonomic instability observed during the ICU stay.

Recent data from a retrospective cohort study support this clinical presentation [3]. Among patients admitted for medically supervised opioid withdrawal, those who were positive for both xylazine and fentanyl had higher systolic blood pressures on day 1 and day 2 compared to those positive for fentanyl alone [3]. This was observed despite no significant difference in Clinical Opiate Withdrawal Scale (COWS) scores or heart rate [3]. These findings are similar to the hospital course observed in this case, marked by persistent hypertension and psychomotor agitation not adequately addressed by sedatives. Hence, xylazine withdrawal may manifest with atypical autonomic features that fall outside the scope of current opioid withdrawal assessment tools, underscoring the urgent need for evidence-based practice guidelines [2,3].

Seizure-like activity in this patient persisted after the administration of levetiracetam in the emergency room and despite sedation and intubation. This prompted the addition of lacosamide. Lacosamide is a selective modulator of slow inactivation of voltage-gated sodium channels [4]. It is primarily indicated for partial and focal seizures [4]. Compared to levetiracetam, lacosamide is metabolized through the liver, making it preferable in this patient with mild AKI [4]. Additionally, it has minimal drug interactions [4]. Lacosamide has been used as an adjunctive treatment in adults with refractory epilepsy [5]. Its intravenous formulation provides a fast onset and favorable central nervous system penetration, making it an effective alternative to counteract the rebound neuroexcitation associated with xylazine withdrawal [4].

As sedation was weaned, the patient was transitioned to buprenorphine to prevent opioid withdrawal, and dexmedetomidine infusion was initiated as soon as xylazine withdrawal was recognized [6]. Dexmedetomidine, a selective alpha-2 adrenergic agonist, is similar to xylazine [6]. It provides sedation, anxiolysis, and autonomic stabilization without respiratory depression, making it an ideal agent for bridging alpha-2 activity in withdrawal [6,7]. However, the transient rise in blood pressure following dexmedetomidine initiation was attributed to initial peripheral alpha-2B receptor activation and delayed central alpha-2A receptor activation, resulting in an early spike in systemic vascular resistance [7].

The key target to address rebound hypertension from xylazine withdrawal is an alpha-2 agonist. Clonidine acts on the posterior hypothalamus and medulla, in contrast to tizanidine, which also functions as an alpha-2 agonist but acts predominantly at the spinal level to induce muscle relaxation with limited systemic effects [8]. As a result, clonidine was selected as the primary pharmacological intervention for blood pressure control in this case [9].

A transdermal clonidine patch was used in the ICU and later was transitioned to oral clonidine with careful titration to maintain alpha-2 support on the medical ward. Adjunctive antihypertensive agents - including amlodipine and losartan - were continued across levels of care and subsequently discontinued, with labetalol and hydralazine administered as needed for rescue therapy.

After the patient was transferred from the ICU to the neuro-telemetry floor, gabapentin was introduced. Gabapentin binds to the α2δ subunit of voltage-gated calcium channels, reducing the release of excitatory neurotransmitters such as glutamate, substance P, and norepinephrine [10]. This mechanism helped mitigate sympathetic rebound and reduced neuropathic pain [10]. Lorazepam was also used to manage persistent agitation and anxiety. It was successfully tapered from intravenous to oral formulation as symptoms improved.

Importantly, the patient’s underlying bipolar disorder required ongoing consideration throughout hospitalization. Psychiatric destabilization in the context of withdrawal can complicate management and prolong recovery. Quetiapine, approved by the Food and Drug Administration (FDA) for bipolar depression, was used in this case [11]. Its antagonism of dopamine D2 and serotonin 5-HT2A receptors, along with its antihistaminergic (H1) and alpha-1 adrenergic blocking activity, contributes to antipsychotic, mood-stabilizing, anxiolytic, and sedative effects [11]. These effects are similar to aripiprazole, which the patient was already taking at home, though quetiapine provides greater sedation and calming benefits due to its mechanism of action [11].

This case underscores the complexity of managing xylazine withdrawal, particularly in the setting of polysubstance use and psychiatric comorbidity. Our multidisciplinary approach - combining alpha-2 agonists, antiepileptics, adjunct antihypertensives, and psychomotor stabilization - provides a foundation for addressing xylazine withdrawal in hospitalized patients (Table 3).

**Table 3:** Summary of key pharmacological interventions in the ICU and in the medical ward. QID: four times a day; BID: two times a day; TID: three times a day

Indications	Medications
Rebound hypertension	In the ICU, dexmedetomidine started with 0.4 mcg/kg/h and peaked at 1.4 mcg/kg/h and was later titrated off. Clonidine transdermal patch started with 0.2 mg/24 hours. In the telemetry unit, the patch was switched to oral clonidine 0.1 mg QID and then reduced to 0.1 mg TID.
Psychomotor agitation and seizure prophylaxis	Lorazepam was started with 2 mg IV BID, then 1 mg IV BID, and then switched to 0.5 mg PO BID. Quetiapine continued as 50 mg PO TID. Lacosamide was started at 50 mg IV BID and then switched to 50 mg PO BID.
Neuropathic pain	Gabapentin was continued at 300 mg PO TID.
Opioid withdrawal prophylaxis	Fentanyl started with 50 mcg/h and peaked at 300 mcg/h and was later titrated off and transitioned to sublingual buprenorphine 2 mg BID.

A key limitation in this case is the lack of routine toxicology screening for xylazine in hospitals, which often leaves clinicians reliant on history-taking and clinical suspicion to recognize xylazine use and withdrawal. Currently, no standardized diagnostic criteria exist, and diagnosis must be based on characteristic findings and exclusion of other etiologies [2]. This highlights the urgent need to incorporate xylazine into routine toxicology panels and to develop formal diagnostic tools to facilitate timely recognition and management [12].

## Conclusions

It is important to recognize potential xylazine withdrawal in addition to opioid withdrawal due to the possibility of worsened vital signs and clinical status. Persistent hypertension, psychomotor agitation, and limited reversibility by naloxone are cardinal symptoms that help distinguish xylazine withdrawal from opioid withdrawal. It is also essential to address coexisting medical conditions to prevent delays in care. Alpha-2 agonists such as clonidine are recommended to treat secondary hypertension resulting from xylazine withdrawal due to their greater systemic impact. Dexmedetomidine may serve as a useful bridging agent for autonomic stabilization during acute withdrawal. Gabapentin and lacosamide have also shown positive outcomes, as seen in this case, by reducing psychomotor agitation associated with reflex neuroexcitatory effects.
